# Accelerating computer vision-based human identification through the integration of deep learning-based age estimation from 2 to 89 years

**DOI:** 10.1038/s41598-024-54877-1

**Published:** 2024-02-20

**Authors:** Andreas Heinrich

**Affiliations:** Department of Radiology, Jena University Hospital – Friedrich Schiller University, Am Klinikum 1, 07747 Jena, Germany

**Keywords:** Machine learning, Databases, Translational research

## Abstract

Computer Vision (CV)-based human identification using orthopantomograms (OPGs) has the potential to identify unknown deceased individuals by comparing postmortem OPGs with a comprehensive antemortem CV database. However, the growing size of the CV database leads to longer processing times. This study aims to develop a standardized and reliable Convolutional Neural Network (CNN) for age estimation using OPGs and integrate it into the CV-based human identification process. The CNN was trained on 50,000 OPGs, each labeled with ages ranging from 2 to 89 years. Testing included three postmortem OPGs, 10,779 antemortem OPGs, and an additional set of 70 OPGs within the context of CV-based human identification. Integrating the CNN for age estimation into CV-based human identification process resulted in a substantial reduction of up to 96% in processing time for a CV database containing 105,251 entries. Age estimation accuracy varied between postmortem and antemortem OPGs, with a mean absolute error (MAE) of 2.76 ± 2.67 years and 3.26 ± 3.06 years across all ages, as well as 3.69 ± 3.14 years for an additional 70 OPGs. In conclusion, the incorporation of a CNN for age estimation in the CV-based human identification process significantly reduces processing time while delivering reliable results.

## Introduction

**Figure 1:**
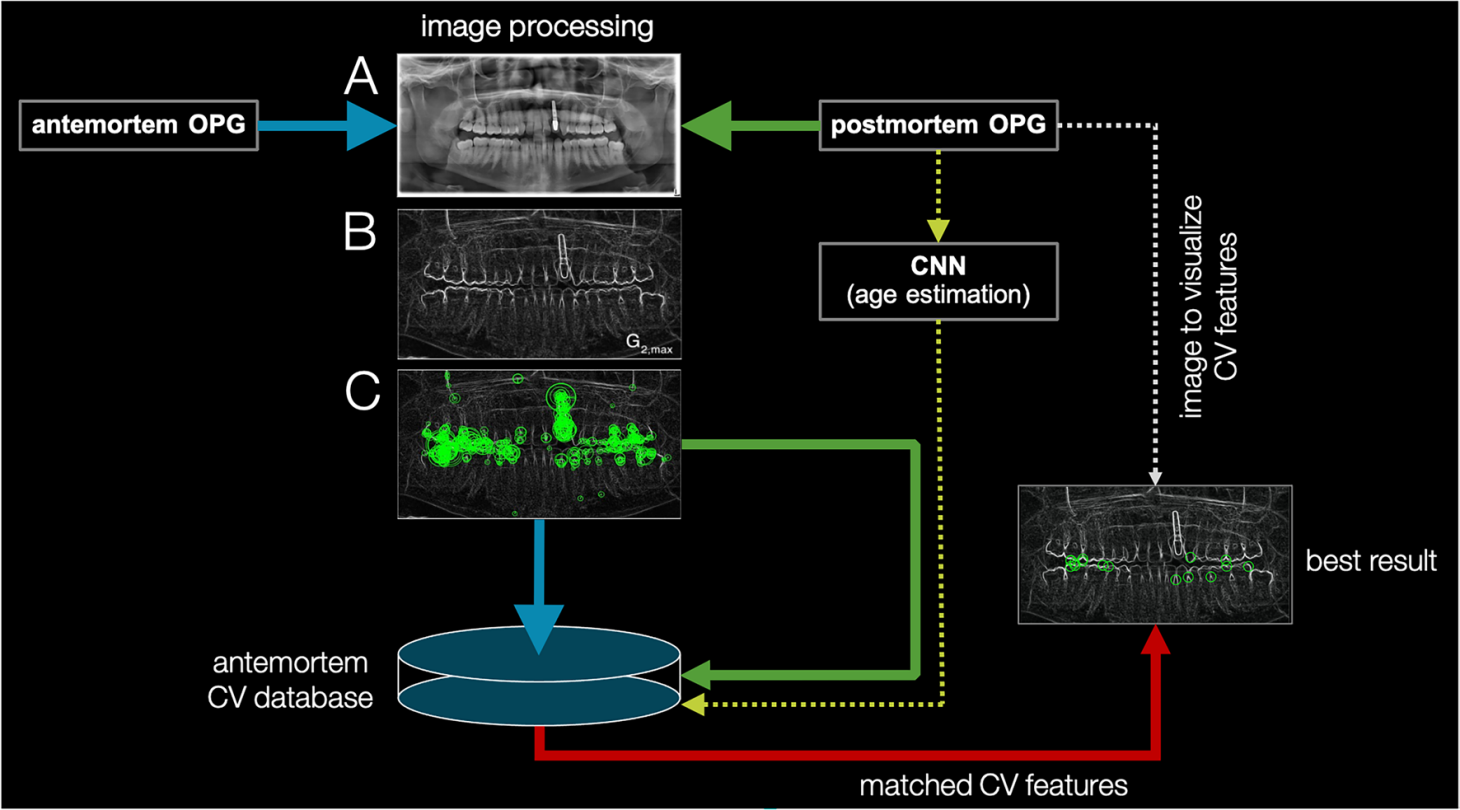
A simplified overview of the C +  + software for CV-based human identification is as follows: Both ante- and postmortem OPGs undergo image processing, involving the following steps. (**A**) The starting point is the original OPG. (**B**) This step includes color depth adjustment, border cropping, edge enhancement using eight 3 × 3 Sobel filters, and image noise reduction through an averaging filter. (**C**) Subsequently, CV features are extracted. The CV features of antemortem OPGs are then stored in the CV database, while the CV features of postmortem OPGs are compared against the CV database, optionally with or without CNN-based age estimation. This process results in matching points between a database entry and the postmortem OPG. If the result with the most matching points ("best result") corresponds to the searched individual, then the identification is considered successful. Additionally, it's possible to analyze, for example, the top 10 results. Dashed lines indicate optional supplementary information.

The international criminal police organization (INTERPOL) designates fingerprints, deoxyribonucleic acid (DNA) profiling, and forensic odontology as primary identification methods due to their scientific reliability^1,2^. INTERPOL has established standardized codes for dental and oral features in postmortem and antemortem images/documents used in identification examinations, enabling the verification of data concordance. The search for suitable antemortem reference materials becomes challenging and time-consuming in the absence of limiting clues to potential identity. A novel Computer Vision (CV)-based method for identifying unknown deceased individuals was introduced in the last few years^3,4^. This method involves extracting CV features from pre-processed images, such as orthopantomograms (OPGs), which are then stored in an antemortem CV feature database (CV database). Automated human identification is achieved by comparing postmortem CV features with an extensive CV database (refer to Fig. 1 and for more details the reference https://www.nature.com/articles/s41598-020-60817-6)^3^. The CV-based human identification is fundamentally not a legally secure method for identification. Its task is rather to locate suitable reference materials and/or obtain clues to the searched individual (refer to supplementary material Fig. S1). By narrowing down the possible identity from thousands of potential identities, a legally secure and forensic identification is subsequently facilitated with appropriate reference materials by a highly qualified professional. This study is part of a project that aims to establish the application of CV-based human identification with very large CV databases.

A CV database can be automatically filled using medical image databases. With the increasing size of a CV database, the signal processing time increases, thus extending the time it takes to identify a individual. Additional filters, such as estimated age of the unknown individual, can help avoid unnecessary queries in the database. Age estimation is a highly complex process that demands expert knowledge. Convolutional Neural Networks (CNNs) have displayed significant promise in objective and automated age estimation, as evidenced by multiple studies focused on OPGs^5–10^. However, there is currently limited experience in CNN-based age estimation for postmortem individuals, and results for adults age groups often fall short of expectations. Furthermore, existing CNNs for age estimation with OPGs have typically been trained, validated, and tested on relatively small datasets, ranging from 304 to 10,400 OPGs for training, 76 to 2,600 OPGs for validation, and 95 to 2,000 OPGs for testing^5,6,8–11^. Therefore, age estimation using CNN and OPGs is frequently implemented based on transfer learning.

Forensic age estimation is based on the developmental stages of certain anatomical structures, such as hand and wrist bones^12^, medial clavicular epiphysis^13,14^, and third molars^15^. In Germany, the Study Group on Forensic Age Diagnostics (AGFAD) of the German Forensic Medicine Society (DGRM) comprises leading experts in the field. They recommend utilizing a maximum diversity of age indicators for age estimations in the living^15–19^. While age estimation in the living is not the primary focus of this study, the cited works illustrate the social, legal, and scientific sensitivity of age estimation as a forensic issue. It should be emphasized that the goal of a fully automated approach for age estimation, as presented in this study, cannot replace forensic age estimation. The approach is intended solely as an auxiliary tool for intelligent searches in CV databases. Therefore, it is not obligated to achieve the same level of accuracy as a forensic science expertise provided by forensic scientists in court. Moreover, the CNN cannot "explain to the judge how it yielded the estimate", which may be a crucial consideration in court.

The primary objective of this study was to develop a standardized and robust CNN for age estimation using OPGs and incorporate it into the method of human identification through a CV-database. To achieve this, a larger dataset comprising 40,000/10,000 OPGs for training/validation and 10,779 OPGs for CNN testing will be leveraged, covering a wider age range from 2 to 89 years. Furthermore, the CNN's applicability for age estimation in cases involving postmortem OPGs will be assessed, and within the context of CV-based human identification for an additional set of 70 independent OPGs divided into 7 age categories.

## Results

### CV-based human identification with CNN for age estimation

Integrating a CNN for age estimation into CV-based human identification with a CV database of 105,251 entries reduced signal processing time by up to 96% (refer to Table 1). The signal processing time was 9.34 ± 9.48 min per individual for ± 5-year search range, 18.64 ± 16.84 min per individual for ± 10-year search range, 57.66 ± 39.41 min per individual for ± 15-year search range, and 246.00 ± 5.47 min per individual without age filtering. Applying the age filter (100% corresponding to the full database with 105,251 entries), only 1–17% (± 5 years), 3–33% (± 10 years) and 5–46% (± 15 years) of CV database rows needed evaluation, based on the individual age. The number of considered rows varies with age and search radius. The CNN was tested with 70 OPGs (10 per age group) that were not part of its training or testing.

**Table 1:** CNN-based age estimation of 70 individuals (not used for training, validation or testing) was applied to reduce the signal processing time for a CV-based human identification process.

Age [years]	Age estimation MAE [years]	Signal processing time per individual [min] (identified with best result = searched individual)			
		± 5 years	± 10 years	± 15 years	All
< 20	2.24 ± 2.48	2.17 ± 0.61 (9/10)	4.11 ± 1.15	7.22 ± 2.93	246.68 ± 4.14
20–29	1.73 ± 0.88	7.28 ± 5.98 (10/10)	12.74 ± 10.59	17.84 ± 15.36	246.17 ± 2.90
30–39	2.69 ± 2.43	9.83 ± 7.96 (8/10)	14.14 ± 10.62	26.43 ± 16.71	244.86 ± 3.19
40–49	4.70 ± 3.05	9.87 ± 7.03 (4/10)	19.46 ± 8.95	63.59 ± 11.63	248.48 ± 3.20
50–59	4.78 ± 4.47	10.17 ± 8.23 (6/10)	31.44 ± 24.71 (9/10)	87.97 ± 15.52	241.22 ± 10.05
60–69	3.98 ± 2.43	11.52 ± 10.17 (7/10)	21.59 ± 9.45	102.65 ± 11.38	247.68 ± 3.78
> = 70	5.71 ± 3.59	14.52 ± 14.85 (4/10)	26.98 ± 21.69 (9/10)	97.91 ± 11.93	246.92 ± 3.71
All	3.69 ± 3.14	9.34 ± 9.48 (48/70)	18.64 ± 16.84 (68/70)	57.66 ± 39.41 (70/70)	246.00 ± 5.47 (70/70)

The search radius was set-based on the estimated age: ± 5, ± 10, or ± 15 years. Without the application of an age filter (refer to the 'all' column), the signal processing time was significantly longer.

The results showed a mean absolute error (MAE) of 3.69 ± 3.14 years and a mean signed error (MSE) of 1.70 ± 4.56 years compared to the actual age (refer to Table 1). In the case of CV-based human identification with the best result being the searched individual (rank 1), 48 out of 70 (69%) were identified within a ± 5-year search range, 68 out of 70 (97%) within a ± 10-year search range, and all 70 out of 70 (100%) within a ± 15-year search range.

### Evaluation the CNN for age estimation

The CNN for age estimation was successful when applied to postmortem OPGs, demonstrating a MAE of 2.76 ± 2.67 years and a MSE of 1.10 ± 4.09 years relative to the actual age. Furthermore, in the antemortem test dataset comprising over 10,779 OPGs, the MAE was 3.26 ± 3.06 years, and the MSE was 0.16 ± 4.47 years across all age groups relative to the actual age (refer to Table 2). The study's findings indicated the attainment of reliable age estimations, with MAE ranging from 1.48 years (95% CI 1.14, 1.82) in the age group of 2–9 years to 6.02 years (95% CI 5.54, 6.50) in the age group of 80–89 years.

**Table 2:** Result of applying the CNN for age estimation to a test dataset that was not used for either training or validation for 10,779 antemortem OPGs and three postmortem OPGs.

Age [years]	OPGs	Absolute error [years]				Success rate [%]			
		MAE	SD	SE	95% CI	± 5 years	± 10 years	± 15 years	± 20 years
2–9	264	1.48	2.83	0.17	1.14, 1.82	97.35	98.48	99.24	99.24
10–19	774	1.44	1.57	0.06	1.33, 1.55	97.80	99.48	99.74	100
20–29	2005	1.99	1.86	0.04	1.91, 2.07	94.06	99.45	99.90	99.95
30–39	1295	3.16	2.68	0.07	3.02, 3.31	79.77	97.45	99.77	100
40–49	1426	3.70	3.11	0.08	3.54, 3.86	73.00	95.30	99.23	100
50–59	1889	3.89	3.26	0.07	3.74, 4.04	71.47	94.81	99.05	100
60–69	1604	3.74	2.99	0.07	3.59, 3.89	73.07	95.32	99.75	100
70–79	1149	4.06	3.28	0.10	3.87, 4.25	68.84	95.13	98.87	99.91
80–89	376	6.02	4.71	0.24	5.54, 6.50	49.20	82.71	94.41	98.94
All	10,782	3.26	3.06	0.03	3.20, 3.32	78.58	96.17	99.30	99.93

The mean absolute error (MAE) gives the magnitude of the deviation between the actual age and the predicted age. The MAE was reported along with the standard deviation (SD), standard error (SE), and 95% confidence interval (CI). The success rate describes the percentage of OPGs in which the age prediction was correct, with an acceptable error range of ± 5, ± 10, ± 15, and ± 20 years.

**Figure 2:**
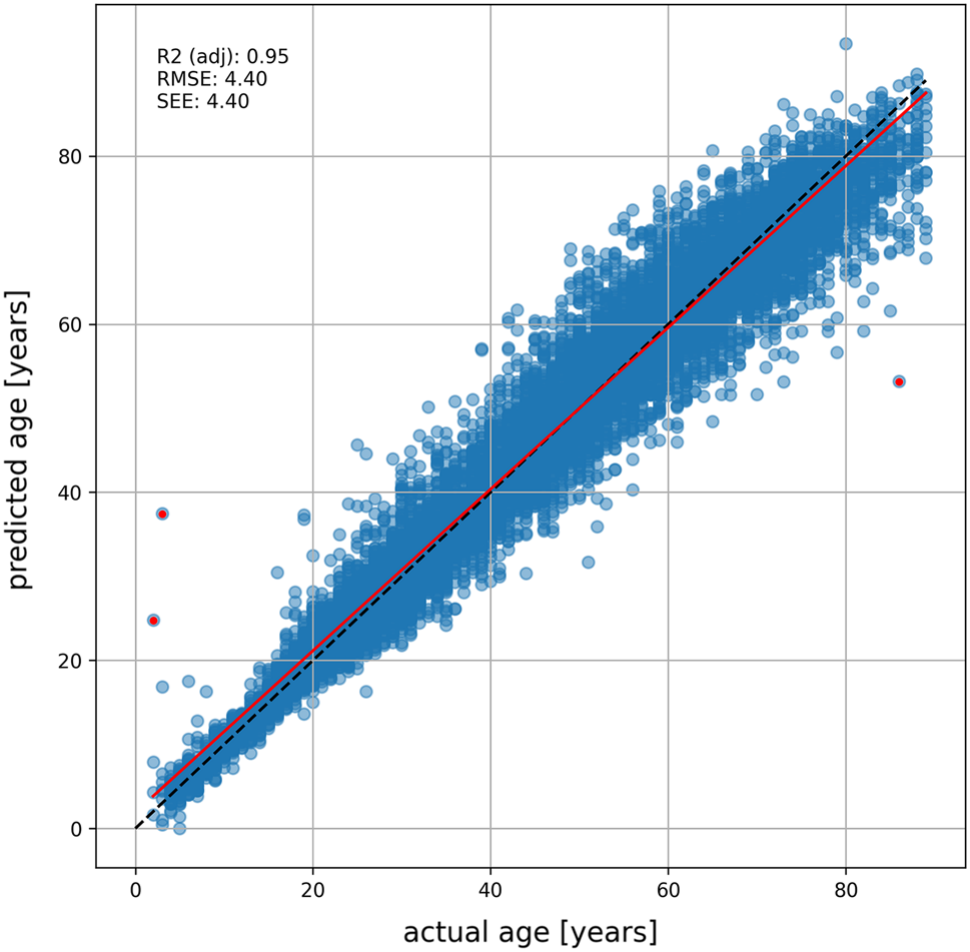
A scatterplot shows the actual and predicted ages for CNN-based age estimation. The dashed black line represents the ideal case, and the red line represents linear regression. The red dots correspond to the OPGs in Fig. 4M–O.

**Figure 3:**
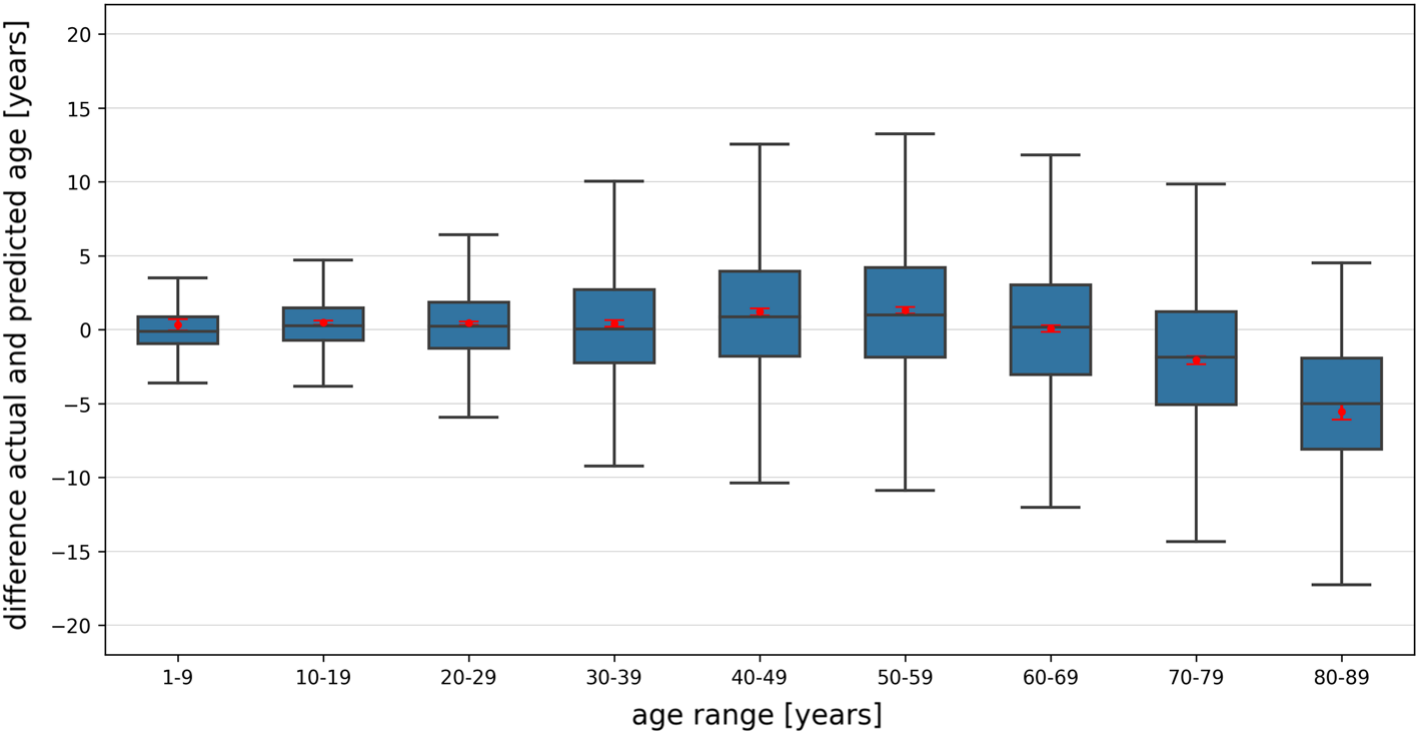
Boxplots display the signed error between actual and predicted age, along with 95% confidence intervals (indicated by red points), for all age groups in CNN-based age estimation. These boxplots illustrate data variability, accounting for factors that can affect age estimation precision, such as measurement errors, image quality, and clinical considerations like pre- and post-surgical imaging. Narrow confidence intervals indicate consistent and reliable age estimations.

The age estimation success rates (age prediction was correct) within various error ranges across all data were as follows: 24.23% within ± 1 year, 43.71% within ± 2 years, 58.91% within ± 3 years, 70.03% within ± 4 years, 78.58% within ± 5 years, 96.17% within ± 10 years, 99.29% within ± 15 years, and 99.93% within ± 20 years. The success rate was highest for the youngest age range and decreased with increasing age. The scatter plot (refer to Fig. 2) and the boxplots (refer to Fig. 3) illustrate a correlation between decreasing prediction accuracy and increasing age, particularly evident from the age of 70 onward. In these cases, individuals were often predicted to be younger than their actual age. The results for each age year are presented in supplementary material Table S1.

**Figure 4:**
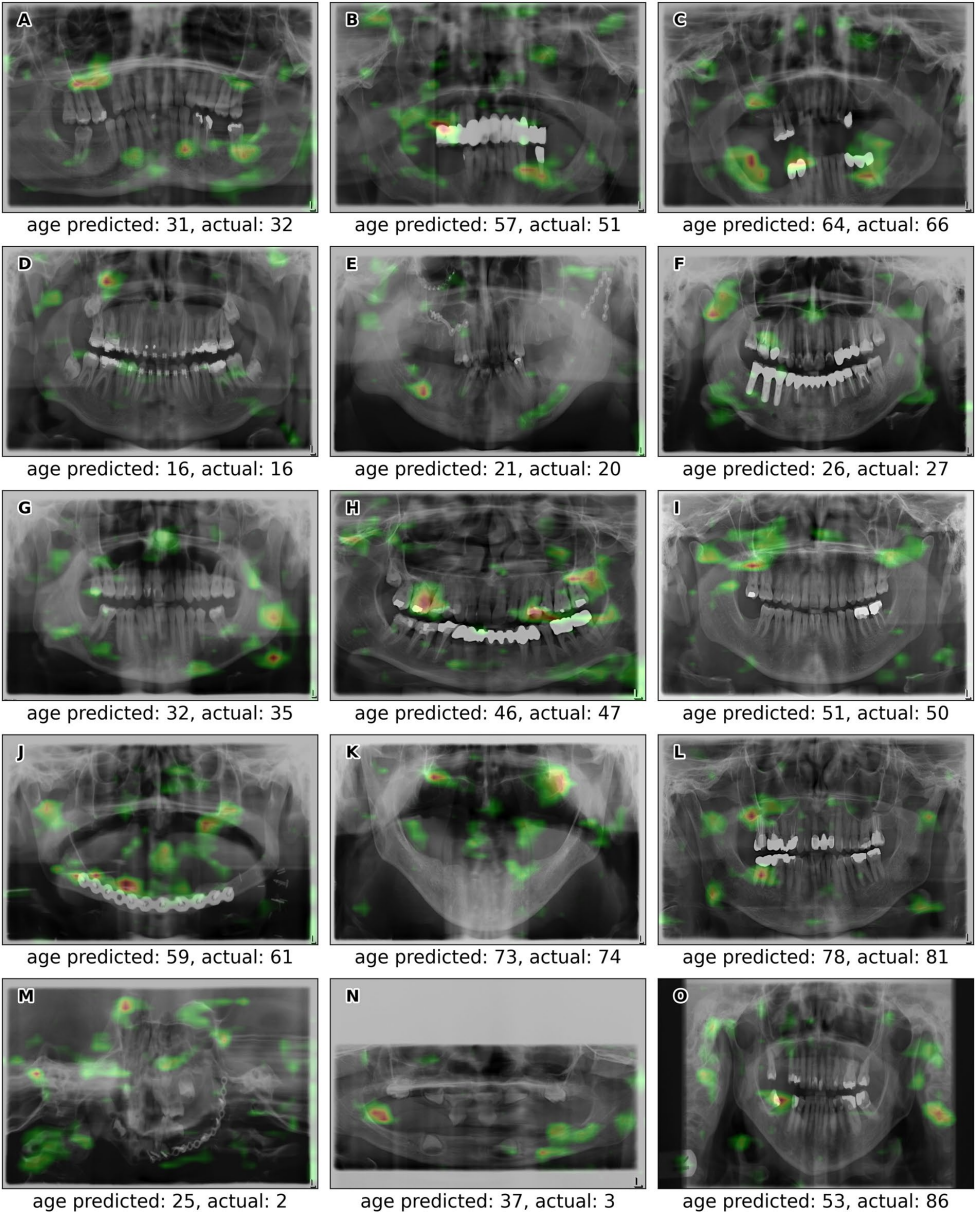
Examples of successful predicted and actual ages for postmortem (**A**–**C**) and antemortem OPGs (**D**–**L**). Additionally, examples of predicted and actual ages for antemortem OPGs with an absolute error exceeding 20 years (**M**–**O**) are marked with red dots in Fig. 2. The OPGs are overlaid with a Grad-CAM representation to highlight the areas of interest, revealing the CNN's focus on specific features important for age estimation.

In Fig. 4 and in supplementary material Fig. S2 without Gradient-weighted Class Activation Mapping (Grad-CAM), examples of OPGs with predicted and actual ages are presented. The absolute error in age estimation exceeded 20 years for only eight out of 10,779 OPGs (0.07%), corresponding to actual ages of 2, 3, 25, 79, 82, 85, 86, and 89 years. Three examples are shown in Fig. 4M–O.

The CNN's performance was assessed with an R^2^ value of 0.95 and a root mean squared error (RMSE) of 4.40 years. When applying the CNN, there was no statistical significance (P > 0.5) observed with respect to sex or whether the individual (not the test OPG) was already known during the CNN training. The CNN was trained for 2216 epochs, resulting in a MAE of 2.80 years and a loss of 12.63 years squared for the training set. In validation, the CNN achieved a MAE of 3.42 years and a loss of 22.06 years squared. The test dataset of 10,779 OPGs have a MAE of 3.26 years and a loss of 19.97 years squared. Using OPGs with different resolutions for the CNN does not reduce age prediction accuracy (refer to supplementary material Fig. S3). In fact, the trained CNN remains robust across various resolutions, ensuring accuracy for its intended purposes.

### Further analyses

With the same number of training epochs, a larger dataset results in reduced loss and MAE (refer to supplementary material Table S2). When applying the test dataset with 10,779 OPGs, the MAE for the best CNN within 100 epochs is 7.00 ± 5.88 years (5,000 OPGs), 5.66 ± 4.90 years (20,000 OPGs), 4.63 ± 4.11 years (35,000 OPGs), and 4.22 ± 3.86 years (50,000 OPGs).

Transfer learning with InceptionV3, incorporating 50,000 OPGs, yielded a MAE of 4.85 ± 4.29 years (loss 41.87 years squared) for the best CNN on the test dataset, which included 10,779 OPGs.

## Discussion

The process of CV-based human identification for an unknown deceased individual can be significantly expedited when additionally using a CNN to estimate the individual's age. The deep learning-based age estimation has shown reliability for individuals between the ages of 2 and 89 years. For the majority of all tested 10,852 OPGs, a ± 5-year tolerance range is sufficient for expedited identification, as it covers the actual age of the searched individual. However, individuals aged 70 and above are increasingly estimated as younger by the CNN than they actually are, which is why the search radius should be expanded to younger ages. In general, the signal processing time is also influenced by the age distribution of the CV database and the computing technology used.

In cases involving unknown deceased individuals, the acquisition of postmortem images may be a necessary step. These images can be compared to antemortem images to narrow down the possible group of individuals. However, manual comparisons by forensics experts are time-consuming and demand high concentration. Furthermore, experts are limited in the maximum number of possible comparisons they can make, whereas computers can efficiently handle millions of data queries. Automated methods for comparing these images with databases can expedite the identification process, assisting forensic pathologists without replacing them. In essence, CV-based human identification aims to narrow down potential identities, making it easier for qualified professionals to retrieve appropriate reference materials for forensic identification. A CV database offers advantages in preserving patient data privacy (CV features do not allow for image reconstruction) and enabling faster identification with reduced database storage requirements. CV features can be extracted from various images, and the choice of OPGs is practical due to their widespread use, including among younger people. This technology has the potential to be as effective as fingerprint or DNA identification methods.

Automated filtering methods are essential for efficient CV-based human identification with an extensive CV database, as matching with millions of entries can be time-consuming. This study focused on automated age estimation using a CNN, as it can be conducted objectively, independently, and significantly faster than an expert's estimation. Furthermore, there is the potential for additional filtering methods like sex estimation^20^, dental segmentation^21^, and geographical region-based filtering, to avoid unnecessary database queries. The longer it takes to identify an unknown deceased individual, the harder it is to find possible clues to the perpetrator. To enhance the identification probability, it is advisable to store CV features for each individual's OPG in the CV database.

Age estimation holds significant importance in various fields, including forensic science, law enforcement and virtopsy, where it provides an objective assessment of an individual's age when such information is lacking^3,6,22–27^. In expert-based forensic age estimation, teeth are crucial. Various dental factors, such as crown formation, mineralization, root growth, eruption sequence, pulp size, loss, calcification, mobility, and microstructure, are considered^8,9^. Wisdom teeth are reliable for estimating ages between 10 and 24 years^7^. However, forensic age estimation in adults is challenging due to limited reliable methods. Dental pulp, alveolar bone loss and third molars are important in adult forensic age estimation, but it's more uncertain due to variability^9,28,29^. The accepted forensic threshold for dental age estimation method in adults from diverse population groups is a standard deviation below ± 10 years^28^. The inherent variability in dental maturity among 95% of the population is approximately ± 1.5–2 years, meaning that it would be theoretically impossible to achieve a higher level of precision^30^. Studies have reported average error rates of around ± 1–2 years for children and approximately ± 5 to ± 14 years for adults^30^.

Manual methods like Demirjian's, Willems', Cameriere's, Nolla's, Smith's, Haavikko's, and Chaillet's, developed for children and subadults, determine chronological age from dental features observed in X-rays^31,32^. Subjective judgment and a wide margin of error remain problematic^9^, as seen in a review^32^ with contrasting results for the same population. Demirjian's method, widely accepted for forensic age estimation in children, has been extensively applied in various populations^32^ based on developmental stages and characteristics of teeth. Studies^28^ on adults using OPGs demonstrated varying standard errors of estimation (SEE) per tooth or group of tooth. For example, the Kvaal et al. method showed SEE ranging from 3.63 to 11.60 years, mean SEE across all publications being 8.59 ± 1.56 years based on a maximum of 200 OPGs. The Cameriere et al. method exhibited SEE ranging from 3.27 to 6.38 years, mean SEE across all publications being 5.13 ± 1.64 years based on a maximum of 606 OPGs. Kvaal et al. method measures pulp, tooth, and root length, as well as root and pulp width at three levels. Cameriere et al. method analyzes pulp and tooth area. These adult-oriented methods are complex and require expertise in measurement techniques.

Age estimation using a CNN introduces a novel approach by incorporating additional features, beyond teeth and tooth roots, for estimation without the need for preprocessing the OPG. Consequently, a relatively low resolution of 256 × 256 pixels proves adequate for achieving excellent results with a CNN. Figure 4's Grad-CAM analysis revealed that the CNN leverages not only dental features but also a broader set of features across the entire head region for age estimation. Therefore, teeth are not necessarily required for age estimation using a CNN, as demonstrated in Fig. 4J,K. Furthermore, cropping OPGs to focus solely on teeth and tooth roots has been found to be detrimental to age estimation accuracy with a CNN—especially at lower resolutions.

A CNN is tailored for a specific task. In this study, a single CNN for age estimation was applied across a wide age range to integrate seamlessly into a CV-based human identification process. The estimated age is utilized exclusively within the automated process and is not presented as an output. The reason is that the use of artificial intelligence (AI) to expedite tasks typically handled by highly skilled professionals necessitates careful consideration. The single CNN integrates age-related changes in subadults (mineralization, eruption) with those in adults (attrition, root resorption, etc.), potentially leading to increased measurement uncertainty. Overestimated or underestimated predictions in the context of CV-based human identification only result in longer signal processing times, with no legal implications. Exploring the use of two separate CNNs for subadults and adults may reduce uncertainties, enhancing our understanding of system behavior. However, these considerations were not the primary focus of this study. In essence, inaccurate age predictions have significant implications in legal and investigative contexts and should only be conducted by highly skilled professionals. For example, overestimating age can misclassify minors as adults, influencing legal decisions and potentially violating rights. Conversely, underestimating age may misidentify adults as minors, jeopardizing legal protection.

In recent literature, transfer learning approaches^6,8–11^ have been primarily employed, with a relatively small number of OPGs used for training and testing the CNN. The overview in Table 3 comprises studies concerning age estimation through CNN and OPGs across a wide age range, but please note that the list is not exhaustive due to the diversity within the literature. The age ranges investigated ranged from 2 to 90 years, with two studies^6,9^ using almost exclusively OPGs of individuals under 30 years old. Ko et al.^8^ utilized the algorithm Darknet-19 for classifying dental age, achieving accuracy rates ranging from ﻿59 to 84% and 49 to 96%-based on the acceptable deviation ranges of ± 5 and ± 10 years, respectively. Atas et al.^10^ tested transfer learning for six different CNNs. The best-performing one was InceptionV3, which was then modified to improve accuracy and speed. Kim et al.^9^ used a custom CNN for extracting image patches and transfer learning (ResNet-152) for predicting age group of the first molar, resulting in a classification of the age into 5 different groups. Vila-Blanco et al.^6^ proposed two transfer learning methods for predicting chronological age, with the best results achieved using DASNet. The datasets used consisted of 84% of individuals aged below 30 years. Alkaabi et al.^11^ evaluates a custom CNN and various transfer learning methods.

**Table 3:** Overview of literature on age estimation using CNN methods with OPGs across a wide age range.

Reference	Year	CNN method	OPGs		Success rate
			Training/validation	test	
This study	2024	Custom CNN with data augmentation; Input layer: 256 × 256; Age range: 2—89 years	40,000/10,000	10,779 + 70 + 3 post-mortem	MAE: 3.26 ± 3.06 RMSE: 4.40 R^2^: 0.95
Ko et al.^8^	2022	Transfer learning (Darknet-19) to categorizes the input images according to the human age; Input layer: 256 × 256; Age range: 20–89 years	13,000	2,000	Classification in 5 groups ± 5 years error age: confusion matrix/table 15–25: 77.30%/84% 25–35: 56.32%/66% 35–45: 53.11%/59% 45–55: 57.87%/67% 55–65: 70.98%/66%
Atas et al.^10^	2022	Transfer learning (VGG16, InceptionV3, Resnet50V2, DenseNet201, MobileNetV2, EfficentNetB4) with data augmentation; Input layer: 256 × 256; Age range: 8–68 years	962/170	200	best-performing InceptionV3 MAE: 3.13 RMSE: 4.77 R^2^: 0.87
Kim et al.^9^	2021	Custom CNN for extracting image patches and transfer learning (ResNet-152) for predicting age group of the first molar with data augmentation techniques; Input layer: 151 × 112; Age range: 0–> 60 years (59% of dataset < 30 years)	1,078/190	318	Classification in 5 groups age: confusion matrix 0–19: 92.31% 20–29: 93.40% 30–39: 38.00% 40–49: 7.89% > 50: 84.38%
Vila-Blanco et al.^6^	2020	Transfer learning (DANet, DASNet) with cross-validation and data augmentation; Input layer: 256 × 128; Age range: 4.5–89.2 years (84% of dataset < 30 years)	1,603/400	286	Best-performing DASNet MAE: 2.84 ± 3.75 years R^2^: 0.90
Alkaabi et al.^11^	2019	Custom CNN and transfer learning (VGG16, VGG19, MobileNet, Inception, Resnet, Alexnet, GoogleNe); Input layer: 224–256 × 224–256; Age range: 2–90 years	1572	678	Test accuracy 29.76–38.27%

Note that this list may not be exhaustive due to the diversity in the literature.

All of these studies^6,8–11^ have in common their use of a low resolution, typically up to 256 × 256 pixels, which has consistently yielded favorable results. As a result, this study also employed a 256 × 256 pixel resolution. Furthermore, these studies utilize transfer learning, advantageous for achieving rapid and satisfactory results with limited datasets. Ad-hoc CNNs may outperform transfer learning when trained specifically for a particular solution^33,34^. Transfer learning, often relying on pretraining with non-medical images, carries the risk of inheriting undesired patterns or biases, potentially diminishing accuracy in addressing specific issues. In this study, the ad-hoc CNN outperformed transfer learning, possibly due to the diversity of OPGs. Notably, no OPG filtering was performed, except for excluding improperly stored images and those from anonymous individuals. While meticulous filtering (e.g., excluding images with large implants, good image quality, no artifacts) can improve results. However, deviating from this specific group might result in less optimal outcomes. Nevertheless, the objective of this study necessitates a diverse range of OPGs to avoid compromising potential CV-based human identification through unreliable age filtering. The emphasis is not on achieving the most accurate age estimation but rather on obtaining a reliable estimation, even with less optimal OPGs. This approach aims to enhance result robustness, potentially even for postmortem OPGs.

The fluctuating number of training samples per age year is sufficiently balanced and has an adequate quantity, excluding the edges of the age range (refer to supplementary material Fig. S4). Additionally, the minor surplus of male OPGs does not significantly impact the CNN's performance in this study (P > 0.5). The developed CNN exhibits robust generalizability due to the diversity of OPGs, allowing effective learning of various patterns. The decision to omit data balancing was made because the methods commonly employed to address imbalanced data in classification may not be the most suitable solution for regression, and further research is needed^35^. Treating different ages as separate groups in regression isn't the most effective because it doesn't make the most of similarities between close ages^35^. Increasing the number of OPGs has had a positive impact on the outcome within the same number of trained epochs. However, there may be a saturation point where additional datasets do not significantly enhance the CNN's effectiveness. Currently, this saturation point has not been reached with the proposed CNN, even with 50,000 OPGs. The decision to use the 2216-epoch CNN was-based on its performance evaluation on a separate validation dataset, which demonstrated a balance between improved performance and the risk of overfitting. To enhance the robustness of the CNN, data augmentation techniques and different resolutions of the OPGs were employed during training.

This study highlights how using a CNN for age estimation can speed up the process of automated CV-based human identification. Further acceleration can be achieved by sorting the database entries for comparison with the postmortem OPG based on the absolute difference from the estimated age. This approach has the potential to successfully identify more than half (52.15%) of the OPGs tested in this study with a search radius of less than 2.5 years. Otherwise, the search radius will automatically expand until the entire database has been queried. In the case of a significant match (where the matching points are significantly higher than typically found between different individuals), the search can be concluded prematurely, saving additional measurement time.

This study has several limitations. The CNN, trained on antemortem OPGs, was tested on only three postmortem OPGs, emphasizing the need for additional investigations to ensure robust evidence due to the limited sample size. Another limitation is the utilization of datasets solely from a single hospital. This research explores the synergy between CV-based human identification and deep learning-based age estimation, revealing advantages for the former. While it does not claim to identify the best CNN for age estimation, it demonstrates the reliable handling of a broad range of OPGs, significantly reducing the duration of CV-based human identification. The study does not constitute research in the context of forensic age estimation or forensic human identification. No forensic expert knowledge is incorporated during the training of the CNN.

In conclusion, this study has shown that a robust CNN for age estimation using OPGs across a wide age range of 2 to 89 years can substantially reduce the signal processing time for automated CV-based human identification. This enables the effective handling of much larger CV databases and can be used in conjunction with other time-saving methods. Using more datasets improves the performance of a CNN for age estimation. The CNN has not yet reached the maximum benefit from an increasing number of training data, even with 50,000 OPGs. An individual CNN tailored for a specific task and trained from scratch can outperform transfer learning, especially when ample datasets are available.

## Methods

All methods used in the study have been approved by the local ethics committee of the Jena University Hospital (registration number 2019-1505-MV) and were carried out in accordance with relevant guidelines and regulations. As this was a retrospective analysis of routine work, written informed consent was waived by the ethics committee.

### CV-based human identification

#### Preprocessing OPGs and CV feature extraction

The original CV-based human identification method used Matlab and the CV algorithm Speeded Up Robust Features (SURF)^3,4^. To enhance efficiency, the method was reimplemented in C +  + with an embedded SQLite database. The new implementation maintains the same preprocessing steps for OPGs before CV feature extraction (refer to Fig. 1 A for the original OPG). First, the color depth was normalized to 8 bits and the margins were cropped to a final size of 180 mm × 100 mm. Then, eight Sobel filter masks (0°–315°, step size 45°) were used to enhance the edges in the OPG, and the edge strength was modulated with a parameter of 1.8. The maxima of the pixels from all eight Sobel gradient images were used to create a single image with emphasized edges in all directions. Afterwards, an averaging filter with a size of 6 was applied to reduce image noise (refer to Fig. 1 B for the ultimate preprocessed OPG). Finally, the CV algorithm AKAZE was employed with the following parameters: Octaves = 8, Layers = 5, Diffusivity = PM_G2, and Descriptor type = KAZE_UPRIGHT, for extracting CV features (refer to Fig. 1C for an example of extracted CV features).

#### Matching CV features with CV database

The matching process generates index pairs that indicate matches between the CV features of the unknown individual and a database entry, resulting in unique matching points. The RANdom SAmple Consensus (RANSAC) algorithm was employed for robustness by removing outliers. The number of remaining matching points indicated identification success. Matching was performed twice, first with the order "unknown individual—database entry" and then with the order "database entry—unknown individual". The average matching points was calculated from these two runs. In this study, CV-based human identification was considered successful if the result with the highest number of matching points corresponded to the searched individual (rank 1).

#### Evaluation CV-based human identification with integrated CNN for age estimation

The CNN for age estimation was integrated into the CV-based human identification software. A random OPG sample of 70 individuals (not used for CNN development process), divided into 7 age categories (10 individuals per category), was used for CV-based human identification with and without CNN-based age estimation. CV features from 105,251 antemortem OPGs of 56,008 individuals were extracted and stored in the CV database. The signal processing time for the CV-based human identification process and the success rate (best match = the searched individual, rank 1) were measured for age estimation error tolerances of ± 5, ± 10, and ± 15 years. The evaluation has been performed on a standard computer (Intel® i5-8500, 6 × 3 GHz CPU, 32 GB 2133 MHz RAM).

### Development of the CNN for age estimation

#### Datasets

**Figure 5:**
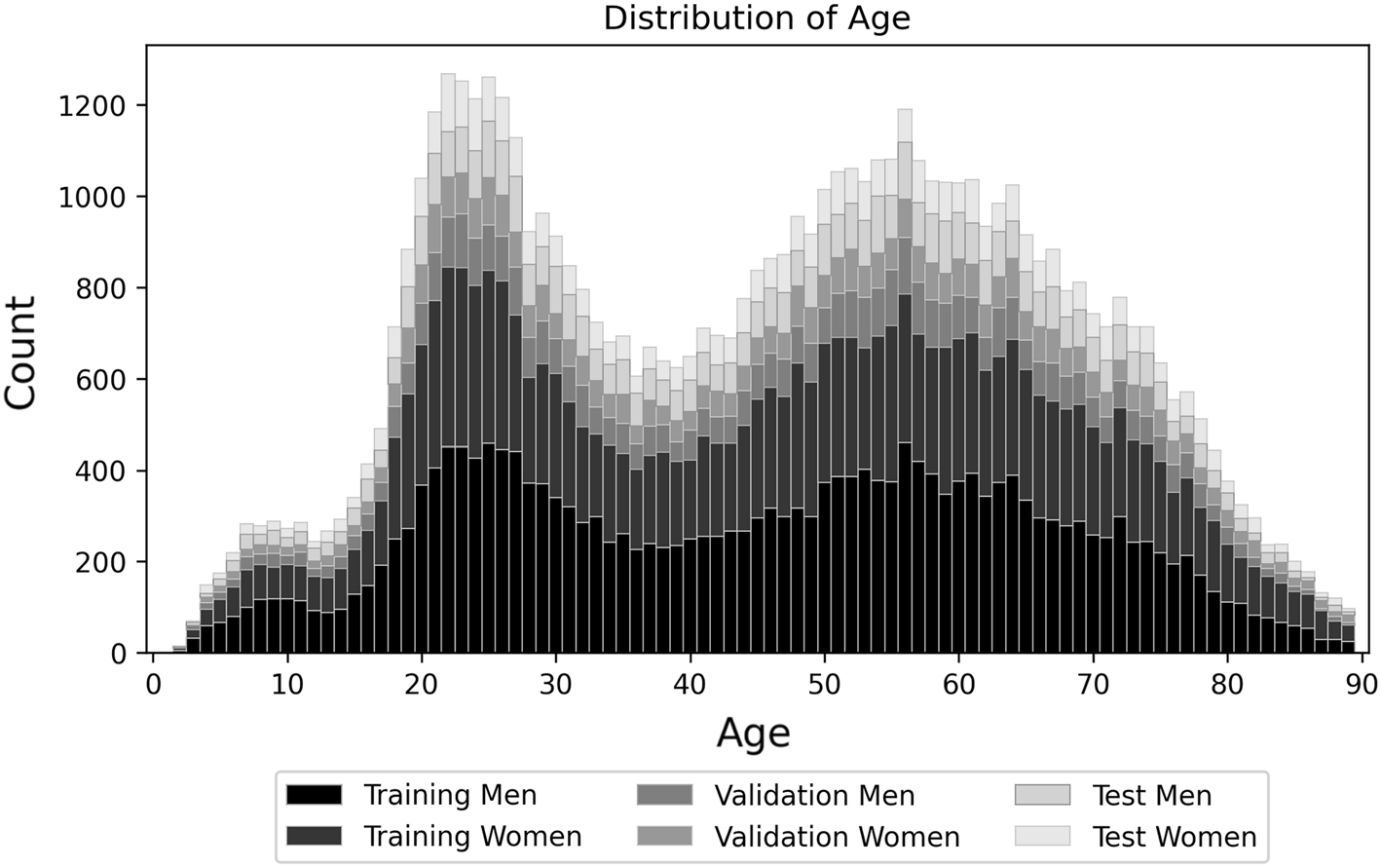
Overview of the used OPGs, divided by age and sex, for training and validation of the CNN (50,000 OPGs), as well as for an application of the CNN (three postmortem and 10,779 antemortem OPGs). The data is represented through stacked bar charts.

For the development and testing of the CNN for age estimation, 60,779 antemortem OPGs and three postmortem OPGs (actual ages 32, 51 and 61 years) were used (refer to Fig. 5), acquired consecutively from 33,045 individuals between 2006 and 2019. A fixation system was utilized for postmortem OPG acquisition, incorporating a mobile bedstead with a sterilizable wooden plate, a metal rail with a slide-on fixation structure, and adjustable elements for securing the body in a seated position, along with provisions for head and teeth support (further details and an illustration can be found in the reference^3^). OPGs that were improperly stored or obtained from anonymous individuals were excluded from the study. The OPGs had a color depth between 8 and 16 bits with different resolutions (in particular, 18,859 OPGs with 2948 × 1540 pixels, 10,731 OPGs with 2440 × 1280 pixels, 7267 OPGs with 2948 × 1552 pixels, 6476 OPGs with 1688 × 875 pixels and 2944 OPGs with 2648 × 1280 pixels). All OPGs were scaled to a size of 256 × 256 pixels.

#### CNN development

Several CNN architectures and hyperparameter configurations, including variations in batch size, learning rate, and dropouts, were systematically evaluated. The study focuses on the most promising CNN selected from these experiments. A dropout rate of 0.25, a batch size of 64, and a learning rate of 0.0001 were chosen as the optimal hyperparameters for the final CNN. The CNN was implemented in python using Keras library.

The final CNN architecture (https://github.com/AG-TraFo/CNN-age-estimation) consists of four blocks of three pairs of convolutional layers and batch normalization each followed by max pooling and dropout layers to extract image features and to avoid overfitting (refer to supplementary material Fig. S5). The number of filters in each blocks of convolutional layers ranges from 32, 64, 128 to 256, with a fixed filter size of 3 × 3. The flattened output from the convolutional layers is fed into a fully connected layer containing 1024 units, followed by batch normalization and dropout layers. For regression tasks, the final layer consists of a single output unit with linear activation. The CNN was compiled using the Adam optimizer and the mean squared error loss function. The MAE was computed as a metric during training.

#### CNN training and evaluation

The CNN was trained for 2500 epochs using a dataset of 50,000 OPGs with corresponding age labels from 2 to 89 years. The data was split into a training set (females/males 18,071/21,929 OPGs) and a validation set (females/males 4,531/5,469 OPGs) with a 80/20 ratio. After every 10 epochs, the training and validation data are thoroughly shuffled separately. In order to increase the diversity and number of training examples, data augmentation was applied using the ImageDataGenerator class in Keras. The datagen object was configured to perform several image transformations during training, including 0–1° rotation, 0–10% width and 0–20% height shifting, 0–20% zooming, and horizontal flipping. The fill_mode parameter was set to nearest, which specifies the strategy used to fill in any newly created pixels during the transformation.

For the age estimation evaluation, the CNN trained for 2216 epochs was used as it produced the most promising results. The CNN was applied to three postmortem OPGs and 10,779 OPGs (refer to Fig. 5). The results were statistically analyzed to investigate deviations and success rates in age categories. In addition, the influence of sex and whether an individual was already known in the training of the CNN were examined using a t-test with significance level of 0.05.

#### Further analyses

To assess the impact of numbers of training OPGs, the CNN underwent training for 100 epochs using datasets of 5,000, 20,000, 35,000, and 50,000 OPGs, respectively. In all sub-datasets, OPGs ranging from 2 to 89 years were included. The best CNN from each training set was then evaluated using the 10,779 OPGs in the test dataset.

Transfer learning with InceptionV3 was performed using 50,000 OPGs. The process involved a batch size of 64, a learning rate of 0.0001, and early stopping with patience set to 10. ImageNet weights were utilized during this phase, with the weights frozen. Additionally, the best CNN from this phase underwent further fine-tuning, during which the weights were unfrozen, using a learning rate of 0.00001.

### Supplementary Information

Supplementary Figures.

Supplementary Tables.

## Data Availability

The datasets analyzed during the current study are not publicly available due to privacy concerns related to the large amount of patient images. However, these datasets are available from the corresponding author on reasonable request, subject to appropriate ethical and legal considerations. The final CNN architecture is accessible at: https://github.com/AG-TraFo/CNN-age-estimation.
