# Supplementary material for: Accelerating computer vision-based human identification through the integration of deep learning-based age estimation from 2 to 89 years
Source: Sci Rep. 2024 Feb 20;14:4195. doi: 10.1038/s41598-024-54877-1 (PMC10879188; doi:10.1038/s41598-024-54877-1)
Supplement: Supplementary file 1 — Supplementary Figures. [file 41598_2024_54877_MOESM1_ESM.pdf]

Accelerating Computer Vision-based Human Identification through the Integration of Deep Learning-based Age Estimation from 2 to 89 years

Andreas Heinrich

Department of Radiology, Jena University Hospital –

Friedrich Schiller University, 07747 Jena, Germany

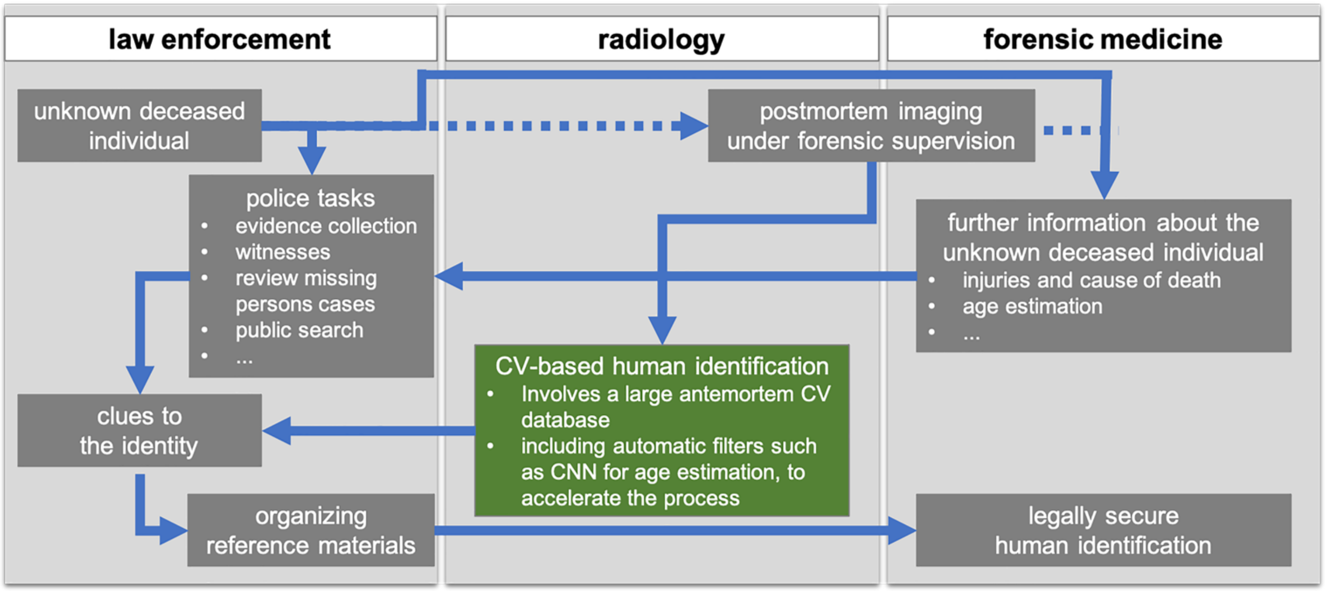

Figure S1 The overview delineates the utilization of a Computer Vision (CV)-based method for identifying unknown deceased individuals. The primary objective is to unveil potential identity clues, thereby organizing reference materials for a legally secure human identification in forensic medicine. Postmortem imaging is presented as an optional step, indicated by a dashed line. Radiology has access to a plethora of antemortem images, contributing to a vast CV database. To bolster intelligent searches in the CV database, this study introduces a CNN for age estimation.

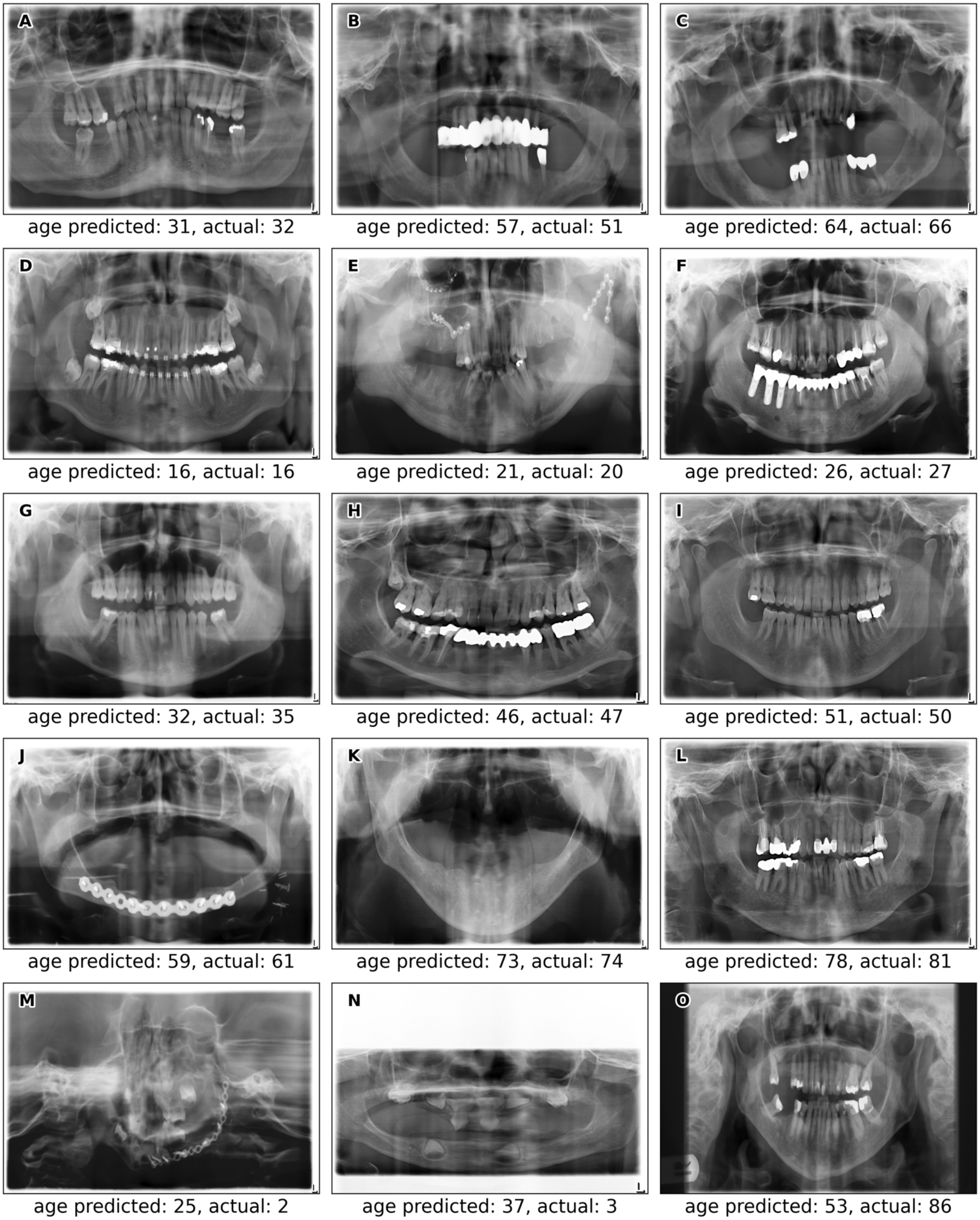

Figure S2 Examples of successful predicted and actual ages for postmortem (A-C) and antemortem OPGs (D-L). Additionally, examples of predicted and actual ages for antemortem OPGs with an absolute error exceeding 20 years (M-O).

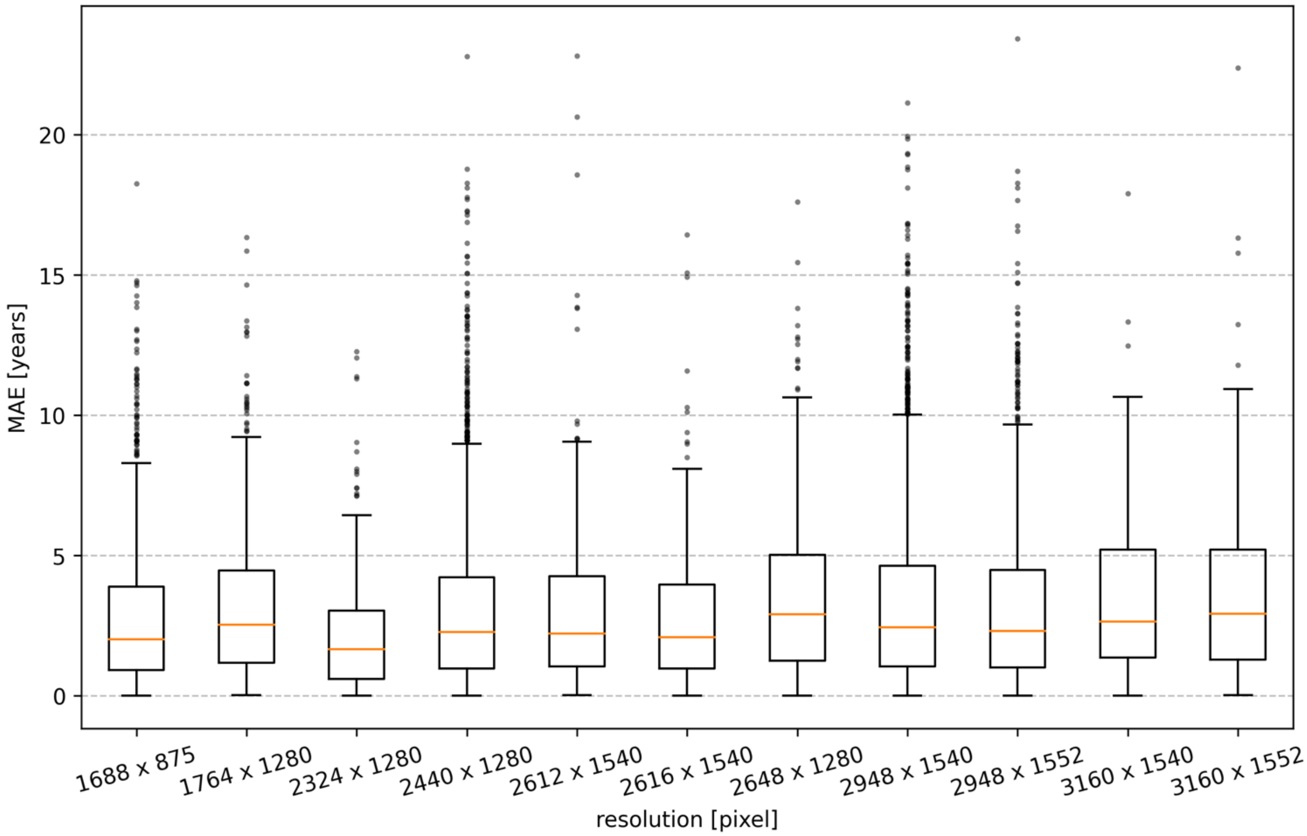

Figure S3 Boxplots show the CNN's performance on a test dataset of 10,779 OPGs with varying resolutions (minimum 150 data points per category). The trained CNN exhibits robustness across different resolutions and provides the required accuracy for its intended purposes.

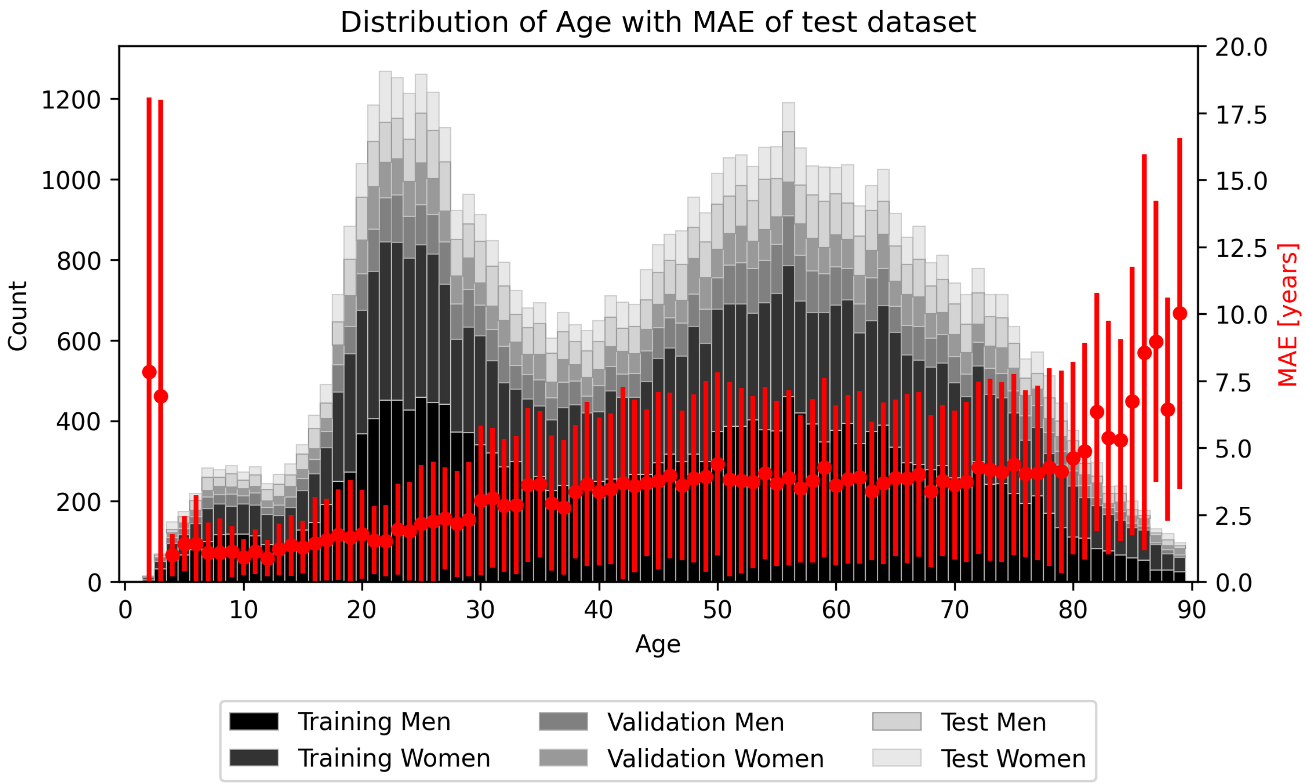

Figure S4 The image displays a combination of Figure 5 and the mean absolute error (MAE) from supplementary material Table S1. The number of training datasets does not have a direct impact on the MAE in a CNN for age estimation through regression.

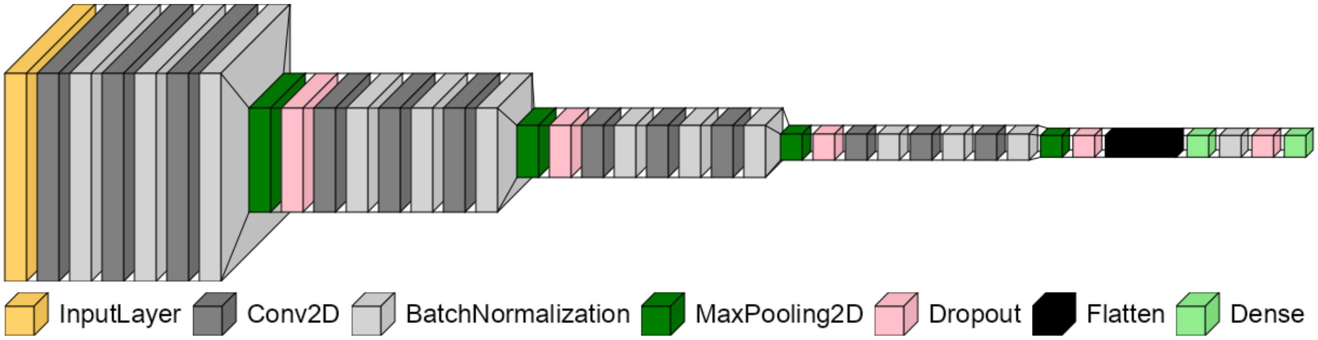

Figure S5 The structure of the CNN is depicted.
