# Supplementary material for: Accelerating computer vision-based human identification through the integration of deep learning-based age estimation from 2 to 89 years
Source: Sci Rep. 2024 Feb 20;14:4195. doi: 10.1038/s41598-024-54877-1 (PMC10879188; doi:10.1038/s41598-024-54877-1)
Supplement: Supplementary file 2 — Supplementary Tables. [file 41598_2024_54877_MOESM2_ESM.pdf]

Accelerating Computer Vision-based Human Identification through the Integration of Deep Learning-based Age Estimation from 2 to 89 years

Andreas Heinrich

Department of Radiology, Jena University Hospital –

Friedrich Schiller University, 07747 Jena, Germany

Age [years]	OPGs	MAE [years]	MAE SD [years]	MSE [years]	MSE SD [years]	successrate [%]			
						± 5 years	± 10 years	± 15 years	± 20 years
**2**	4	7.84	10.23	7.65	10.43	50.00	75.00	75.00	75.00
**3**	9	6.93	11.07	5.92	11.71	77.78	77.78	88.89	88.89
**4**	26	0.99	0.79	0.30	1.25	100.00	100.00	100.00	100.00
**5**	29	1.42	1.03	-0.17	1.77	100.00	100.00	100.00	100.00
**6**	43	1.42	1.80	0.50	2.24	97.67	97.67	100.00	100.00
**7**	57	1.09	1.12	-0.30	1.54	98.25	100.00	100.00	100.00
**8**	42	1.08	1.28	-0.07	1.68	97.62	100.00	100.00	100.00
**9**	54	1.13	0.96	-0.03	1.49	100.00	100.00	100.00	100.00
**10**	41	0.92	0.67	-0.09	1.14	100.00	100.00	100.00	100.00
**11**	48	1.12	0.83	0.02	1.40	100.00	100.00	100.00	100.00
**12**	44	0.88	0.69	-0.19	1.11	100.00	100.00	100.00	100.00
**13**	55	1.21	0.96	-0.32	1.52	100.00	100.00	100.00	100.00
**14**	56	1.36	1.14	-0.26	1.77	98.21	100.00	100.00	100.00
**15**	63	1.29	0.98	0.17	1.62	100.00	100.00	100.00	100.00
**16**	84	1.43	1.74	0.47	2.21	98.81	98.81	100.00	100.00
**17**	85	1.57	1.53	0.86	2.02	95.29	100.00	100.00	100.00
**18**	126	1.75	1.69	1.14	2.15	96.03	99.21	100.00	100.00
**19**	172	1.65	2.15	0.81	2.59	96.51	98.84	98.84	100.00
**20**	190	1.77	1.65	0.76	2.31	95.26	99.47	100.00	100.00
**21**	204	1.55	1.27	0.53	1.93	97.06	100.00	100.00	100.00
**22**	226	1.53	1.34	0.35	2.00	98.67	100.00	100.00	100.00
**23**	202	1.96	1.70	0.67	2.51	94.55	99.50	100.00	100.00
**24**	218	1.89	1.84	0.56	2.58	94.50	99.54	100.00	100.00
**25**	220	2.17	2.19	0.52	3.04	94.55	99.09	99.55	99.55
**26**	216	2.26	2.23	0.21	3.18	93.52	99.07	99.54	100.00
**27**	208	2.36	1.90	0.39	3.01	91.35	99.04	100.00	100.00
**28**	162	2.16	1.98	0.00	2.94	91.36	99.38	100.00	100.00
**29**	159	2.33	2.13	0.34	3.15	87.42	99.37	100.00	100.00
**30**	171	3.03	2.79	0.66	4.07	81.29	95.32	100.00	100.00
**31**	164	3.13	2.61	0.41	4.06	79.88	98.17	100.00	100.00
**32**	146	2.85	2.47	0.22	3.77	83.56	98.63	100.00	100.00
**33**	129	2.86	2.57	0.59	3.81	89.15	97.67	99.22	100.00
**34**	115	3.61	2.87	0.72	4.56	73.04	95.65	100.00	100.00
**35**	128	3.64	2.72	0.25	4.54	71.88	96.88	100.00	100.00
**36**	109	2.93	2.52	0.43	3.85	81.65	99.08	100.00	100.00
**37**	117	2.77	2.51	0.06	3.74	83.76	98.29	100.00	100.00
**38**	100	3.35	2.49	0.43	4.16	76.00	98.00	100.00	100.00
**39**	116	3.65	3.07	0.45	4.76	75.00	97.41	98.28	100.00
**40**	121	3.37	2.75	0.98	4.25	77.69	96.69	100.00	100.00

**41**	126	3.47	2.81	0.50	4.45	75.40	94.44	100.00	100.00
**42**	122	3.69	3.59	1.52	4.93	75.41	94.26	97.54	100.00
**43**	122	3.58	3.23	1.31	4.65	74.59	95.90	98.36	100.00
**44**	149	3.69	2.74	0.99	4.50	73.83	97.32	100.00	100.00
**45**	148	3.77	3.32	1.75	4.71	69.59	95.27	98.65	100.00
**46**	151	3.96	3.10	0.80	4.97	70.86	92.72	100.00	100.00
**47**	173	3.59	2.79	0.93	4.46	71.10	96.53	100.00	100.00
**48**	153	3.85	3.14	1.38	4.78	73.20	95.42	100.00	100.00
**49**	161	3.93	3.56	1.74	5.01	70.81	94.41	97.52	100.00
**50**	189	4.40	3.42	1.93	5.23	64.55	93.65	98.94	100.00
**51**	189	3.82	3.63	0.83	5.21	74.60	93.12	97.88	100.00
**52**	177	3.77	3.47	0.91	5.05	72.32	93.22	98.87	100.00
**53**	186	3.72	3.21	1.43	4.71	72.58	94.09	99.46	100.00
**54**	205	4.06	3.21	1.83	4.84	69.27	94.63	99.51	100.00
**55**	175	3.67	3.08	1.79	4.45	75.43	96.57	98.29	100.00
**56**	198	3.88	3.27	0.96	4.99	68.69	95.96	98.99	100.00
**57**	189	3.48	2.76	1.15	4.30	77.25	97.35	100.00	100.00
**58**	181	3.77	3.06	0.85	4.79	72.38	94.48	100.00	100.00
**59**	200	4.26	3.34	1.25	5.27	68.50	95.00	98.50	100.00
**60**	167	3.59	2.99	1.26	4.50	71.86	95.21	100.00	100.00
**61**	186	3.83	3.15	0.46	4.95	74.19	93.55	99.46	100.00
**62**	173	3.90	3.23	0.71	5.02	72.83	94.80	100.00	100.00
**63**	162	3.39	2.58	0.11	4.27	75.31	98.77	100.00	100.00
**64**	161	3.68	3.02	0.09	4.77	73.91	95.65	100.00	100.00
**65**	168	3.88	2.93	-0.02	4.87	72.02	97.62	98.81	100.00
**66**	143	3.86	3.16	-0.49	4.98	69.93	93.01	100.00	100.00
**67**	175	4.01	3.07	0.12	5.05	72.00	93.71	99.43	100.00
**68**	127	3.38	2.83	-1.08	4.29	78.74	96.06	100.00	100.00
**69**	142	3.78	2.83	-1.04	4.62	70.42	95.07	100.00	100.00
**70**	128	3.61	2.79	-0.86	4.49	75.00	98.44	99.22	100.00
**71**	144	3.72	2.99	-1.59	4.51	72.92	95.83	99.31	100.00
**72**	139	4.26	3.21	-1.42	5.15	64.75	93.53	99.28	100.00
**73**	129	4.18	3.39	-2.28	4.89	68.99	93.80	98.45	100.00
**74**	132	4.10	3.35	-1.69	5.03	66.67	94.70	98.48	100.00
**75**	104	4.38	3.38	-2.18	5.10	62.50	94.23	99.04	100.00
**76**	90	4.03	3.12	-2.64	4.38	67.78	94.44	100.00	100.00
**77**	92	4.06	3.26	-2.59	4.53	69.57	95.65	100.00	100.00
**78**	107	4.27	3.69	-3.34	4.56	68.22	94.39	97.2	100.00
**79**	84	4.11	3.78	-3.21	4.58	71.43	96.43	97.62	98.81
**80**	65	4.62	3.59	-3.64	4.60	61.54	87.69	100.00	100.00
**81**	60	4.88	4.05	-4.35	4.62	61.67	86.67	98.33	100.00

**82**	51	6.34	4.45	-6.13	4.74	41.18	86.27	96.08	98.04
**83**	41	5.38	4.36	-4.77	5.04	58.54	87.80	95.12	100.00
**84**	40	5.28	3.77	-4.60	4.59	55.00	85.00	100.00	100.00
**85**	37	6.74	5.01	-6.61	5.18	40.54	83.78	91.89	97.30
**86**	20	8.56	7.39	-8.30	7.69	40.00	75.00	80.00	95.00
**87**	18	8.97	5.25	-8.78	5.59	16.67	66.67	77.78	100.00
**88**	30	6.44	4.17	-6.25	4.45	36.67	76.67	96.67	100.00
**89**	14	10.02	6.55	-10.02	6.55	28.57	50.00	71.43	92.86

Table S1 The CNN for age estimation was applied to a test dataset comprising 10,779 antemortem OPGs and three postmortem OPGs. This dataset had not been utilized during either the training or validation phases. The evaluation metrics included the mean absolute error (MAE) and mean signed error (MSE), both accompanied by their respective standard deviations (SD). Additionally, the success rate was calculated, representing the percentage of OPGs for which the age prediction fell within acceptable error ranges of ±5, ±10, ±15, and ±20 years.

	**CNN**	**CNN**	**CNN**	**CNN**	**InceptionV3**	**InceptionV3 fine-tuning**	**best CNN**
Training OPGs	5,000	20,000	35,000	50,000	50,000	50,000	50,000
best epoch	94	98	95	97	49	27	2216
validation dataset							
MAE [years]	7.73	5.48	4.74	4.43	9.93	5.24	3.42
Loss [years^2]	102.82	54.17	41.77	36.50	158.33	49.28	22.06
test dataset with 10,779 OPGs							
MAE [years]	7.00 ± 5.88	5.66 ± 4.90	4.63 ± 4.11	4.22 ± 3.86	9.99 ± 7.48	4.85 ± 4.29	3.26 ± 3.06
MSE [years]	-0.69 ± 9.11	0.88 ± 7.44	0.23 ± 6.18	0.88 ± 5.65	3.04 ± 12.10	-0.34 ± 6.46	0.16 ± 4.47
Loss [years^2]	83.51	56.08	38.30	32.71	155.71	41.87	19.97

Table S2 For a further analysis, the CNN in this study was trained for 100 epochs with varying numbers of training OPGs (80/20 ratio for training/validation). Additionally, transfer learning was performed using InceptionV3, and the best CNN underwent further fine-tuning. For comparison, the results for the best CNN in this study are also presented. The CNNs were evaluated based on mean absolute error (MAE), mean signed error (MSE), and loss.
